# Review of the Geant4-DNA Simulation Toolkit for Radiobiological Applications at the Cellular and DNA Level

**DOI:** 10.3390/cancers14010035

**Published:** 2021-12-22

**Authors:** Ioanna Kyriakou, Dousatsu Sakata, Hoang Ngoc Tran, Yann Perrot, Wook-Geun Shin, Nathanael Lampe, Sara Zein, Marie Claude Bordage, Susanna Guatelli, Carmen Villagrasa, Dimitris Emfietzoglou, Sébastien Incerti

**Affiliations:** 1 Medical Physics Laboratory, Department of Medicine, University of Ioannina, 45110 Ioannina, Greece; demfietz@uoi.gr; 2 Department of Accelerator and Medical Physics, Institute of Quantum Medical Science, QST, Chiba 263-8555, Japan; sakata.dousatsu@qst.go.jp; 3 Bordeaux University, CNRS/IN2P3, CENBG, UMR 5797, 33170 Gradignan, France; ngochoang.tran.vn@gmail.com (H.N.T.); zein@cenbg.in2p3.fr (S.Z.); incerti@cenbg.in2p3.fr (S.I.); 4 Laboratoire de Dosimétrie des Rayonnements Ionisants, IRSN, Institut de Radioprotection et de Sûreté Nucléaire BP17, 92262 Fontenay-aux-Roses, France; yann.perrot@irsn.fr (Y.P.); carmen.villagrasa@irsn.fr (C.V.); 5 Department of Radiation Oncology, Seoul National University Hospital, Seoul 03080, Korea; ukguen@gmail.com; 6 Independent Researcher, Melbourne, VIC 3000, Australia; nathanael.lampe@gmail.com; 7 Universite Toulouse III-Paul Sabatier, UMR 1037, CRCT, 31034 Toulouse, France; marie-claude.bordage@inserm.fr; 8 Centre for Medical Radiation Physics, University of Wollongong and Illawarra Health and Medical Research Institute, Wollongong, NSW 2522, Australia; susanna@uow.edu.au

**Keywords:** Monte Carlo, Geant4-DNA, DNA damage, DNA repair, mechanistic modeling, track-structure, radiobiological modelling, IRT, step by step

## Abstract

**Simple Summary:**

A brief description of the methodologies to simulate ionizing radiation transport in biologically relevant matter is presented. Emphasis is given to the physical, chemical, and biological models of Geant4-DNA that enable mechanistic radiobiological modeling at the cellular and DNA level, important to improve the efficacy of existing and novel radiotherapeutic modalities for the treatment of cancer.

**Abstract:**

The Geant4-DNA low energy extension of the Geant4 Monte Carlo (MC) toolkit is a continuously evolving MC simulation code permitting mechanistic studies of cellular radiobiological effects. Geant4-DNA considers the physical, chemical, and biological stages of the action of ionizing radiation (in the form of x- and γ-ray photons, electrons and β^±^-rays, hadrons, α-particles, and a set of heavier ions) in living cells towards a variety of applications ranging from predicting radiotherapy outcomes to radiation protection both on earth and in space. In this work, we provide a brief, yet concise, overview of the progress that has been achieved so far concerning the different physical, physicochemical, chemical, and biological models implemented into Geant4-DNA, highlighting the latest developments. Specifically, the “dnadamage1” and “molecularDNA” applications which enable, for the first time within an open-source platform, quantitative predictions of early DNA damage in terms of single-strand-breaks (SSBs), double-strand-breaks (DSBs), and more complex clustered lesions for different DNA structures ranging from the nucleotide level to the entire genome. These developments are critically presented and discussed along with key benchmarking results. The Geant4-DNA toolkit, through its different set of models and functionalities, offers unique capabilities for elucidating the problem of radiation quality or the relative biological effectiveness (RBE) of different ionizing radiations which underlines nearly the whole spectrum of radiotherapeutic modalities, from external high-energy hadron beams to internal low-energy gamma and beta emitters that are used in brachytherapy sources and radiopharmaceuticals, respectively.

## 1. Monte Carlo Radiation Track Simulations

The Monte Carlo (MC) technique is currently recognized as the gold standard in clinical dosimetry of mega-voltage electron and photon beams and a number of MC-based software packages have been developed and become commercially available for patient treatment planning in radiotherapy applications (e.g., PEREGRINE, DPM, VMC++, MCV, MMC, ORANGE) [1]). These dose calculation software programs are based on so-called general-purpose MC radiation transport codes, such as, MCNP, EGS, GEANT, FLUKA, and PENELOPE [2,3,4,5,6]. However, the standard versions of all these MC dosimetry codes have a typical spatial resolution of the order of 0.1 mm that is many orders of magnitude higher than the DNA diameter (nanometer-scale) which is considered the primary radiation target. Consequently, predictions of radiotherapy outcomes in patient treatment planning are presently limited to macroscopic radiation dose calculations in millimeter-size volumes [7]. With radiation biology increasingly focusing on effects at the (sub) cellular and DNA level, the spatial resolution of MC dosimetry codes is important to reach the (sub) micro- and nano-meter scale.

The spatial resolution of MC codes is dependent upon the energy cut-off limit of electron transport. Commercial MC codes simulate electron tracks by using the condensed-history technique which “groups” many interactions along artificial steps and is valid (or recommended) above 1 keV. To reach the (sub) cellular and DNA level, it is necessary to simulate electron transport down to much lower energies approaching the ionization threshold of the medium (about 10 eV for tissue-like materials) by the so-called track-structure approach whereby electron tracks are simulated interaction-by-interaction. 

However, the transition from macroscopic (condensed-history) to microscopic (track-structure) MC simulation requires much more detailed physics models than are commonly available. This has led to the development of several specialized low-energy MC codes (e.g., NOREC [8], PARTRAC [9], KURBUC [10], see Table 1). These codes require a large amount of input data for simulating every radiation interaction of both the primary and secondary particles, which makes them difficult to operate at clinical energies and/or include information from complex irradiation geometries. As a general remark, although track-structure codes eliminate the problem of step-size artifact by avoiding the procedure of introducing artificial step lengths along the track that affect both the resolution and the accuracy of the results, their physical data (i.e., their interaction cross sections) are much more uncertain compared to those that are used in condensed-history codes. In addition, all of the aforementioned track-structure codes are the propriety of the authors and are not publicly distributed. It must be highlighted that the transition from macroscopic (condensed-history) to microscopic (track-structure) simulation, offers not only improved spatial resolution but entirely new potentials. While macroscopic simulation of radiation doses requires the knowledge of the dose-response curve (usually obtained from independent experiments) to predict the biological (therapeutic) outcome, the microscopic simulation may extend to the biological stage of radiation action (albeit at molecular level) and, therefore, to predict some biological effects (and related RBE) a priori, without the knowledge of dose-response curves.

Low-energy electron transport (sub-1 keV energies) is practically unimportant in clinical dosimetry which mostly deals with macroscopic volumes at the tissue (mm) and organ (cm) level. Nonetheless, such electrons are essential in studies of radiation quality and RBE using nano- or micro-dosimetric methods. Mechanistic studies of cellular radiobiological effects are also very much dependent upon the transport of low energy electrons owing to their role in radiolysis which contributes to the indirect damage of DNA [11]. Furthermore, efforts towards biologically-optimized (e.g., RBE-based) patient treatment planning in hadron therapy (as well as other modalities [7]) usually employ microdosimetry concepts [12]. Macroscopic MC codes that are based solely on the condensed-history technique are, in principle, inappropriate for such applications. Therefore, in recent years, there have been efforts to implement track-structure capabilities into general-purpose MC codes to enable energy deposition calculations at the (sub) cellular and DNA level and establish connection with radiolysis models. Notable examples are MCNP (version MCNP6 [13]), Geant4 (including Geant4-DNA extension [14]), PENELOPE (modification to PENELOPE/penEasy [15]), and PHITS [16]. Apart from MCNP6, which uses a simple interpolation of high-energy atomic models down to the eV energy range, thus, neglecting aggregation effects which influence the scattering cross sections of atoms when these atoms form chemical bonds in molecules or in condensed media, the low-energy capabilities of PENELOPE, GEANT4, and PHITS are based on elaborate physics models that are specifically developed for liquid water [4,6,16].

**Table 1:** MC track-structure codes that are used in various radiation effects studies in biological medium. Associated particles, energy ranges, and target media (e.g., whether vapor-v-or/and liquid-l-phase cross sections are used) are indicated. The degree of sophistication of the models differs for each code and it will not be further analyzed in this study.

Code	Particles	Energy Range	Target Materials	Chemical Stage	Reference
CPA100	e^−^	Thermalization–256 keV	Water (l), DNA	Yes	Terrisol and Beaudré (1990) [17]
DELTA	e^−^	≥10 eV–10 keV	Water (v)	Yes	Zaider et al. (1983) [18]
EPOTRAN	e^−^, e^+^	≥7.4 eV–10 keV	Water (l,v)	No	Champion et al. (2012) [19]
ETRACK	e^−^, p, α	≥10 eV–10 keV	Water (v)	Yes	Ito (1987) [20]
ETS	e^−^	≥10 eV–10 keV	Water (l,v)	Yes	Hill and Smith (1994) [21]
Geant4-DNA	e^−^, p, H, α, ions	Thermalization–1MeV e^−^, 100 eV–100 MeV p, H, 1 keV–400 MeVα, 0.5MeV/u−10^6^MeV/u ions	Water (l), DNA, Gold	Yes	Incerti et al. (2010, 2018), Bernal et al. (2015) [14,22,23,24]
IONLYS/IONLYS-IRT	e^−^, p, ions	0.2 eV–150 keV e^−^, p, 0.1 MeV-300 MeV ions	Water (l)	Yes	Cobut et al. (1998) [25]
KAPLAN	e^−^	≥1–10 keV	Water (l,v)	Yes	Kaplan (1990) [26]
KITrack	e^−^, ions	≥10 eV–100 keV	Water (l)	No	Wiklund et al. (2011) [27]
KURBUC (KURBUC/LEAHIST/LEPHIST/CHEM-KURBUC)	e^−^, p, α, C	10 eV–10 MeV (10keV, liq.) e^−^,1 keV–300 MeV p, 1keV/u-2MeV/u α, 1 keV/u–10 MeV/u carbon	Water (l,v)	Yes	Nikjoo et al. (2016) [10]
LEEPS	e^−^, e^+^	0.1–100 keV	All materials	Yes	Fernández-Varea et al. (1996) [28]
LEPTS	e^−^, e^+^, p	Thermalization–10 keV e^−^, Thermalization–10 MeV p	Water (v), CH_4_, C_2_H_4_, C_4_H_8_O, SF_6_, C_4_H_4_N_2_	No	Sanz et al. (2012), Blanco et al. (2013) [29,30]
Lion Track	e^−^, p, ions	>50 eV e^−^, 0.5 MeV/u–300 MeV/u p, ions	Water (l)	No	Bäckström et al. (2013) [31]
MC4	e^−^, ions	≥10 eV e^−^, ≥0.3 MeV/u ions	Water (l,v)	No	Emfietzoglou et al. (2017) [32]
MOCA8B	e^−^	10 eV–100 keV	Water (v)	Yes	Paretzke (1970) [33]
NASIC	e^−^	Thermalization–1 MeV e^−^	Water (l)	Yes	Li et al. (2015) [34]
NOTRE DAME	e^−^, ions	≥ 10 eV e^−^, ≥0.3 MeV/u ions	Water (l,v)	Yes	Pimblott et al. (1990) [35]
OREC/NOREC	e^−^	7.4 eV–1 MeV e^−^	Water (l)	No	Semenenko et al. (2003) [8]
PARTRAC	e^−^, e^+^, p, H, α, ions	1 eV–10 MeV e^−^, 1 keV–1 GeV p, H, α, 1 MeV/u–1 GeV/u ions	Water (l), DNA	Yes	Friedland et al. (2003) [36]
PITS04	e^−^, ions	≥ 10 eV e^−^, ≥ 0,3 MeV/u ions	Water (l)	No	Wilson et al. (2004) [37]
PITS99	e^−^, ions	≥ 10 eV e^−^, ≥ 0,3 MeV/u ions	Water (v)	Yes	Wilson and Nikjoo (1999) [38]
PTra	e^−^, p, α	1 eV–10 keV e^−^, 1–10 MeV α, 300 keV-10 MeV p	Water (l,v), DNA	No	Grosswendt and Pszona (2002) [39]
RITRACKS/RETRACKS	e^−^, ions	0.1 eV–100 MeV e^−^, 10^−1^MeV/u–10^4^MeV/u ions	Water (l,v)	Yes	Plante and Cucinotta (2009) [40]
SHERBROOKE	e^−^, ions	≥ 10 eV e^−^, ≥ 0,3 MeV/u ions	Water (l,v)	Yes	Cobut et al. (2004) [41]
STBRGEN	e^−^, ions	≥ 10 eV e^−^, ≥ 0,3 MeV/u ions	Water (l,v)	Yes	Chatterjee and Holley (1993) [42]
TILDA-V	e^−^, p, H, ions	≥ 7,4 eV e^−^, 10 keV/u–100 MeV/u ions	Water (l,v), DNA	No	Champion et al. (2005) [43]
TRAX	e^−^, p, ions	1 eV–few MeV e^−^, 10 eV–few hundred MeV/u ions	Water (v)	Yes	Krämer and Kraft (1994) [44]
RADAMOL (TRIOL/STOCHECO)	e^−^, ions	≥7.4 eV–2 MeV e^−^, ≥0.3–200 MeV/u ions	Water (l)	Yes	Bigildeev and Michalik (1996) [45]
TRION	e^−^, ions	≥10 eV e^−^, ≥0.3 MeV/u ions	Water (l,v)	No	Lappa et al. (1993) [46]
TRACEL/RADYIE/RADIFF	e^−^, ions	≥10 eV e^−^, ≥0.3 MeV/u ions	Water (l,v)	Yes	Tomita et al. (1997) [47]

## 2. The Geant4-DNA Extension

Since 2007, Geant4 (release 9.1) is the only open access general-purpose radiation transport MC code offering, through its Geant4-DNA low-energy extension, track-structure capabilities in liquid water down to the eV energy range [22]. Liquid water has been historically the medium of choice in track-structure codes because of its abundance in cells (70–80% by weight) and also because of its role as a source of reactive free radicals [48]. Towards more realistic modelling of direct damage to DNA, several studies have presented interaction cross sections that are specific to DNA bases (or constituents) in the gas phase [49,50,51,52,53,54] some of them being part of the Geant4-DNA ongoing developments. Interaction cross sections that are specific to bulk DNA in the condensed-phase have also been presented based on the dielectric approach [55,56,57]. 

Geant4-DNA offers the possibility to transport interaction-by-interaction electrons, protons, hydrogen atoms, alphas, and some ions in liquid water medium. Due to the importance of low- and moderate-energy electrons (from few eV to 1 MeV) in track-structure simulations, as well as the large uncertainties that are associated with their transport in biological media, users have the possibility to select among three recommended sets of alternative physics models which correspond to different cross sections for elastic and inelastic scattering. These physics models are the default Geant4-DNA models or Option 2 constructor (available since 2007 in Geant4 version 9.1) [24], the improved models that were developed at the University of Ioannina [58] or Option 4 constructor (available since 2015 in Geant4 version 10.2), and the Option 6 constructor (available since 2017 in Geant4 version 10.4) [14,22,23,24]. The Geant4-DNA models have been tested and validated against reference data (i.e., NIST, ICRU) and wherever available versus experimental data and other MC simulation studies. Comparisons between the condensed history models of Geant4 and the track-structure models of Geant4-DNA have also been undertaken for particular applications [14,23,59,60,61,62,63].

## 3. Physical Interactions in Geant4-DNA: Energy-Loss Models

Energy-loss models in track-structure simulations determine the spatial distribution of ionizations and excitations, thus, the energy deposition processes taking place within the target volumes. Measured ionization and excitation cross section data for liquid water do not exist (contrary to the case of vapor water). Therefore, theoretical models play an important role in the development of energy-loss models for liquid water and a variety of approaches have been adopted, ranging from some well-established atomic/molecular models [64] to more complicated solid-state models [65,66]. However, the existence of spectroscopic data for several materials (including liquid water) concerning their absorption spectrum or their optical dielectric constants provides an indirect means to calculate inelastic scattering cross sections from first principles using the dielectric theory. Different analytic parameterizations of the dielectric response function [67,68] are currently being used in track-structure codes, such as the NOREC, PARTRAC, KURBUC, and GEANT4-DNA codes. In the framework of the plain wave Born approximation (PWBA), the dielectric response function completely determines inelastic scattering through the proportionality:

(1) d2σBorndE dq∝Im[−1ε(E,q)]

where *E* is the energy transfer, *q* is the momentum transfer (or scattering angle), σ is the cross section, and Im[−1/ε] = Im[ε]/|ε|^2^ is the energy-loss function (ELF). Due to its analytic properties, a Drude-like model of ε(*E*,*q*) is being used in all the above track-structure codes (NOREC, PARTRAC, KURBUC, GEANT4-DNA), which mostly differ on the values of the Drude coefficients and the details of the parameterization of experimental data. The advantage of a Drude representation of ε(*E*,*q*) against using more sophisticated models (e.g., Mermin) has been discussed elsewhere [66].

In Geant4-DNA Option 2 constructor, the experimental data for the imaginary part of the dielectric response function of liquid water [69] are partitioned to the outer ionization shells and excitation levels using the following parameterization [58]:

(2) Im[ε(E,q=0)]=∑nioniz.[Dn(E;En)Θ(E−Bn)]+∑kexcit.[Dk*(E;Ek)Θ(E−Bk)]

where the index *n* runs over the ionization shells and the index *k* runs over the discrete excitation levels, *D_n_*(*E;E_n_*) and D_k_*(*E;E_k_*) are the ordinary and derivative Drude functions with coefficients that were determined by a fit to the experimental data at the optical limit (*q* = 0) under the constraint of the sum-rules, and *B_n,k_* are threshold energies (e.g., binding energies). In Geant4-DNA Option 4 constructor [58], Equation (2) is replaced by

(3) Im[ε(E,q=0)]=∑nioniz.{[D(E;En)−D(E;Bn)exp(Bn−E)+Fn(E)]Θ(E−Bn)}+∑kexcit.{[Dk*(E;Ek)+Fk(E)]Θ(E−Bk)}

where *F_n,k_*(*E*) are analytic functions that are calculated by the new algorithm that is implemented in Option 4 that improve the consistency of the model ELF and facilitate the analytic calculation of the real part, Re[ε], via the Kramers-Kronig relation [58]. Despite using the same experimental optical data set with Option 2 as input, as well as the same ionization shells and excitation levels, substantially different ionization and excitation cross sections are obtained in Option 4 at low energies due to the implementation of the Emfietzoglou-Kyriakou partitioning algorithm [58]. This resulted in higher ion-pair energies (the so-called “W-values”), smaller penetration distances, and less diffused dose-point-kernels [58,70] while also influencing the calculated yields of direct DNA damage [71,72]. In addition, Option 4 includes some methodological changes for a more consistent implementation of the Coulomb and Mott corrections which quantitatively account for most of the exchange-correlation effect that is encountered in electron-electron interactions [73,74]. These corrections are applied to address more accurately the low energy electron inelastic cross sections which have a strong influence on modeling radiobiological effect sat the sub-cellular level.

An alternative to Option 2 and Option 4 constructors is the Option 6 constructor which corresponds to a re-engineering of the CPA100 track-structure code [75]. The original CPA100 code, which was a well-established code in microdosimetry, was developed and maintained by Michael Terrissol and co-workers [17] to simulate the step-by-step transport of electrons and photons in liquid water and different biomolecular targets, such as DNA bases and sugar-phosphate groups [76] in homogeneous as well as heterogeneous materials. CPA100 code can generate all the electronic and photonic cascades (Auger electrons, X-rays, and atomic reorganization). It can also simulate physicochemical and chemical stages during the early passage of particles in matter up to one microsecond to evaluate early DNA damage. In the last version of CPA100 code, the energy differential and the total cross sections of the five molecular orbitals are calculated using the BEB (Binary Encounter Bethe) model [64]. The BEB model is an exchange–corrected atomic model which requires only the knowledge of three physical parameters for each orbital: the binding energy, the mean kinetic energy, and the orbital occupation number. The first advantage of this model is that the cross sections are calculated analytically. The second advantage of the analytical form of the differential cross section is that the energy loss can be obtained by directly sampling the differential cross section expression without using interpolation in very large cross section tables. This is done via a composition sampling method that was originally developed in the last version of CPA100 [77]. The excitation cross sections for the five discrete levels are calculated in the first Born approximation using the optical data model of the complex dielectric response function coming from the work of Dingfelder et al. [78]. This model is also based on a Drude representation of ε(*E*,*q*) using the same optical dataset, electronic excitation levels, and dispersion relations that are similar to the other available electronic models in the Geant4-DNA constructors (Option 2 and Option 4). However, the resulting excitation cross sections are not the same due to a different set of Drude coefficients. Finally, in the Option 6 set of physics models, angular deflection in inelastic scattering for both excitation and ionization is considered based on the kinematics of binary collisions. 

## 4. The Physico-Chemical and Chemical Stages of Water Radiolysis

After ionization or excitation in an energy deposition process, water molecules can dissociate or decay into new reactant species (e^−^_aq_, H_2_, H^•^, ^•^OH, H_3_O^+^, …). These new molecular species can diffuse and interact amongst themselves, producing other molecules. Consequently, after the physical interactions, the number of molecules of a given species evolves in time. During this process, free radicals are created and may react with biological molecules such as DNA, RNA, proteins, etc., and finally, induce early damage. In Geant4-DNA, since version 10.1, the physico-chemical and chemical stages of water radiolysis were introduced [79] that allow simulations of the production, chemical reactions, and transport of reactive species along the passage of radiation up to 1 µs. These two stages, which technically belong to the chemistry module of Geant4-DNA, are described below in more detail.

### 4.1. The Physico-Chemical Stage

In Geant4-DNA, the physico-chemical stage includes the thermalization process of secondary electrons and electronic events that are occurring in ionized and excited water molecules up to 1 picosecond (ps). The primary or secondary electrons that are produced during the physical stage continue to lose their energy through the dissociative attachment and vibrational excitation processes, get thermalized within 110 femtoseconds (fs), and then become solvated within about 250 fs [11,80,81]. The electronic holes of ionized water molecules quickly join proton transfer processes that are occurring between them or a nearby water molecule to create H_3_O^+^ and ^•^OH molecules or participate with solvated electrons in the recombination process which reforms water molecules. The excited water molecules go through dissociation channels which depend on their excited level. The hot dissociation fragments of water molecules and electrons are assumed to become all thermalized within 1 ps. Table 2 presents the decay channels that are implemented in the default physico-chemical stage of Geant4-DNA [80].

### 4.2. The Chemical Stage

#### 4.2.1. Step by Step Method

**Figure 1:**
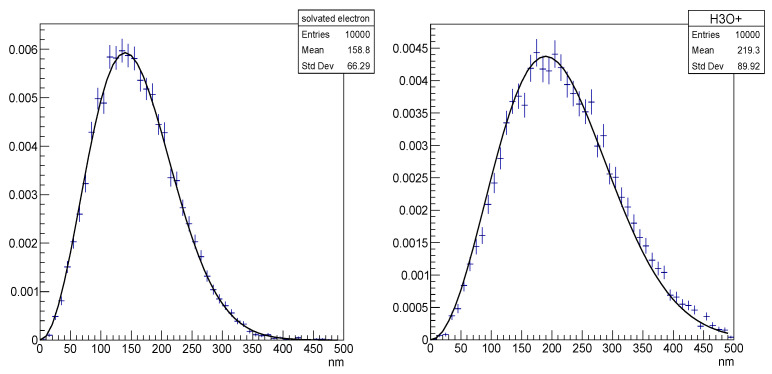
Diffusion range of chemical species (left plot: solvated electron; right plot: H_3_O^+^) set in Brownian motion in liquid water within 1 μs simulated with Geant4-DNA (crosses) compared to the theoretical Smoluchowski solution (line).

From 1 picosecond (*ps)* up to 1 microsecond (*μs*) during the so-called chemical stage, the chemical species can diffuse through the medium and interact with each other. The diffusion process corresponds to the Brownian motion which is described by the solution of the Smoluchowski equation. This well-known solution of the Smoluchowski equation can be given by:

(4) $$p\left(r,\Delta t|r_{0}\right)=\frac{4\pi {\left(r-r_{0}\right)}^{2}}{{\left(4\pi D\Delta t\right)}^{3/2}}e^{\left\{\frac{-{\left(r-r_{0}\right)}^{2}}{4D\Delta t}\right\}},$$

where $r_{0}$ is the initial position and r is the possible next position of free species for the probability $p\left(r,\Delta t|r_{0}\right)$ in a time interval Δt. This probability depends on the diffusion coefficient D in water for each species type. Equation (4) means that the determination of the species positions in Brownian motion is randomly based on a 3D-Gaussian distribution and can be applied only in a space and a time interval Δt where species are free. Figure 1 shows the distributions of displacements that were obtained by a sampling method in Geant4-DNA at 1 *μs*, compared with the distribution that was calculated by Equation (4). The Geant4-DNA sampling method uses a generator of random numbers ($R_{x},\;R_{y},\;R_{z}$) which are normally distributed with zero mean and unit variance to compute the position of the molecule at time t+Δt as:

(5a) $$x\left(t+\Delta t\right)=x\left(t\right)+R_{x}\sqrt{2D*\;\Delta t}$$

(5b) $$y\left(t+\Delta t\right)=y\left(t\right)+R_{y}\sqrt{2D*\;\Delta t}$$

(5c) $$z\left(t+\Delta t\right)=z\left(t\right)+R_{z}\sqrt{2D*\;\Delta t}$$

In the diffusion model that was described earlier, only the molecules of interest are explicitly simulated and the solvent (water) is considered as a continuum. Under these conditions, free species diffuse until two reactants encounter each other. Let’s consider two reactants *A* and *B* in encounter, they will first form a complex (*A:B*) that might either re-dissociate into *A* and *B* or react and create new products *P*. The chemical equation is:

(6) $$A+B\overset{k_{C},\;k_{D}}{↔}\left(A:B\right)\overset{k_{R}}{\to }P$$

where $k_{C}$ is the reaction rate constant of the (*A:B*) complex formation, $k_{D}$ is the dissociation rate constant of the (*A:B*) complex, and $k_{R}$ the activation reaction rate constant. If the reaction is very effective, the reactants will form the new products as soon as the molecules encounter each other. This is described by a very high activation reaction rate constant $k_{R}$≫$k_{C}$,$k_{D}$ (in other words, $k_{R}$→∞). This allows us to define the reaction rate k as:

(7) $$k=\frac{k_{C}}{1-\frac{k_{D}}{k_{R}}}\approx k_{C}$$

These reactions are named diffusion-controlled reactions and are considered to be very effective and occurring immediately when molecules encounter each other. The chosen criterion is usually the separation distance between the reactants; when two reactants are under a certain threshold, the reaction occurs. This threshold is calculated from the reaction rates by the Smoluchowski theory in case of particles without charge:

(8) $$k=4{\pi N}_{A}D$$

and by the Smoluchowski–Debye theory for electro-static interactions:

(9) $$k=4{\pi N}_{A}D\sigma_{eff}$$

where $\sigma_{eff}=\frac{R_{c}}{\exp\left(\frac{R_{c}}{\sigma }\right)-1}$, $R_{c}$ is the Onsager distance, σ is the reaction radius,$\;D=D_{A}+D_{B}$ with *D_A_* and *D_B_* are the diffusion coefficients of reactants A and B in water, and $N_{A}$ is the Avogadro number. Table 3 provides the implemented chemical reactions and corresponding reaction rates of Geant4-DNA (from Geant4 version 10.7). 

To avoid losing potential reactions, time intervals of the species in diffusion are usually defined by discretized small steps (or time step) [84]. However, to maintain the computational efficiency of the model, the time steps should not be too small [80]. Therefore, to optimize the computation time, improved time step models are necessary. 

In Geant4-DNA, since version 10.1, the Step by Step (SBS) approach which uses the dynamic time step model*,* has been implemented. A detailed description of this method can be found elsewhere [79,84]. This method briefly proposes a time step model which allows the definition of virtual time steps during which the reaction cannot occur with at least 95% (default value) of confidence. One can visualize that as creating a protection domain surrounding the particle, ensuring that this particle will not react with any other particle with 95% confidence up to its border. Therefore, within this protection domain, the particle is considered approximately independent (or “free”) and can take longer diffusion time steps. Equation (4) is used to determine the dynamic time step. This process is repeated many times until a chemical reaction takes place. Thus, we may have one- or many-time steps before the reaction occurs. To avoid the scenario of too many small time steps, the Minimum Time Steps and the Brownian bridge approaches have been added to limit the number of time steps to an encounter. While Minimum Time Steps constrain the minimum time-step that is allowed for each reactant pair, the Brownian bridge technique computes the probability of encounter during their Minimum Time Steps and thus compensates for the “missed” reactions. The use of these Minimum Time Steps in Geant4-DNA can be decided by users. 

**Figure 2:**
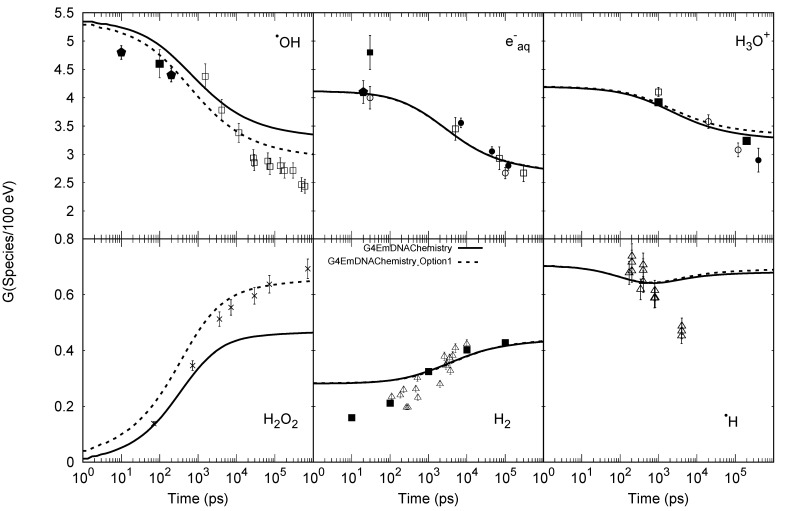
The G-values as a function of time calculated using the two chemistry constructors “G4EmDNAChemistry” and “ G4EmDNAChemistry_option1” of Geant4-DNA, and experimental data: OH^●^: ▯ Laverne, 2000 [85], ■ Jay-Gerin et al., 2000 [86], ⬟ El Omar et al., 2011 [87]; e^−^_aq_: ▯ Shiraishi et al., 1988 [88], ■ Sumiyoshi and Katayama, 1982 [89], ◯ Hunt et al., 1973 [90] and Wolff et al., 1973 [91], ● Buxton, 1972 [92], ⬟ Muroya et al., 2005 [93]; H_3_O^+^: ▯ Pikaev et al., 1977 [94], ■ Cercek and Kongshaug, 1969 [95], ◯ Anderson et al., 1985 [96], ● Schmidt and Ander, 1969 [97]; H_2_O_2_: × LaVerne, 2000 [85]; H_2_: Δ Draganic and Draganic, 1975 [98], ■ LaVerne and Pimblott, 1991 [99]; H^●^: Δ Draganic and Draganic, 1972 [100].

Figure 2 shows the time-dependent radiolytic yields G-values using the *Minimum Time Step* at 1 *ps* given by the two chemistry constructors “G4EmDNAChemistry” and “G4EmDNAChemistry_option1” of Geant4-DNA. While the “G4EmDNAChemistry” constructor, that is based on the PARTRAC MCTS code [36] was available in Geant4 since version 10.1, the “G4EmDNAChemistry_option1” including diffusion coefficients and chemical reaction rates from the RE-/RITRACKS MCTS code [40] was integrated since version 10.5. Both chemistry constructors currently use the SBS approach. The radiolytic yield G-value is defined as the number of molecules at a given time for each 100 eV of deposited energy. In this simulation, Geant4 version 10.7 is used with 80 keV incident electrons irradiated from the center of a very large water volume (see Geant4-DNA example “chem5”) and the minimum and maximum energy depositions set at 1 keV et 2 keV, respectively [11]. A general agreement of these results with experimental data can be observed. It should be noted that the last two parameters are used for energy threshold selection to restrict such energy deposition in a small segment of the entire physical track. While the minimum energy deposition limits the energy loss of the primary particle, the event having total energy deposition larger than the maximum energy deposition will be aborted and is fully ignored in the simulation.

#### 4.2.2. Independent Reaction Time 

Although the dynamic time step model is used to optimize the choice of the time step, the calculation time remains the main drawback of the SBS approach when the simulations deal with a large number of species [101,102]. To avoid this drawback, a more efficient method called *Independent Reaction Time* (IRT) has been implemented in Geant4-DNA in the version 10.7 of Geant4. By simplifying the multiple particle problem to the two-particle problem in an approximation, the IRT method proposes that possible reactant pairs are scheduled independently and organized in a reaction queue sorted by their time to reaction (or “reaction time”). The reactions having the shortest reaction time are then processed successively until no more reaction is in the reaction queue, or the reaction times are longer than the end time of the simulation. 

**Figure 3:**
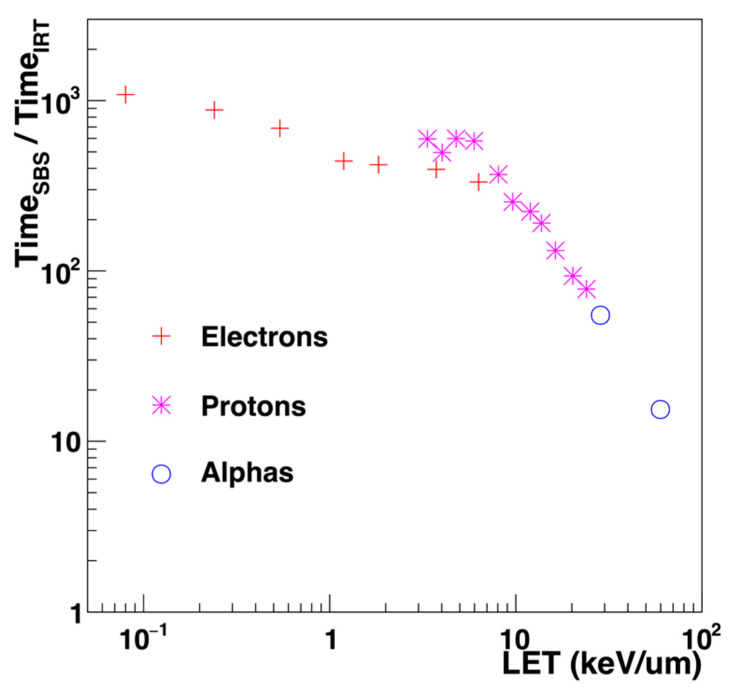
Speedup factor of the SBS approach versus IRT as a function of LET, for electrons, protons, and alphas [106].

The reaction times are sampled according to the reaction probability, which is calculated by the solution of the Smoluchowski equation (4) with boundary conditions (for totally or partially diffusion-controlled reactions) transformed to radial Green’s function in a spherical coordinate system. The details of the IRT are found elsewhere in [100,103,104,105]. This approach allows the performance of water radiolysis simulation with reasonable computational time. Such time depends strongly on the linear energy transfer (LET) of the irradiation source as shown in Figure 3. The IRT model can reduce the simulation time up to 1000 times compared to the SBS method for low-LET simulations as, for instance 1 MeV electrons. Since the search algorithm of the IRT method is less efficient at the high density of chemical species, the ratio of the calculation efficiency between SBS and IRT decreases as a function of LET. Nevertheless, the simulation time for the chemical stage is remarkably reduced using the IRT model.

## 5. Towards the Modelling of Early DNA Damage

### 5.1. History

It is recognized that DNA is the privileged target to be taken into account to understand and predict the consequences of irradiation at the cellular level [107]. DNA damages are categorized as single-strand breaks (SSB), DSBs, base damage, and complex damage when a combination of those is produced. Among the different types of DNA damage, double strand breaks (DSBs) are considered the most deleterious and can be correlated with clonogenic cell kill [108]. The more complex the initial damage to DNA, the higher the probability of being mis-repaired and thus consequences on cell fate can be expected. Therefore, modelling of early damage to DNA represents a powerful tool to predict irradiation risk [109]. This is only possible if a detailed understanding of the mechanisms of ionizing radiation action on living organisms is implemented in the code.

To this end, MC track-structure codes have been developed to describe molecular interactions leading to direct and indirect effects which are at the origin of early DNA damage. The pioneering works have evolved from the consideration of direct effects alone [110,111] to the inclusion of indirect damage [112,113,114]. A key point in these simulations is the geometrical description of the target molecule, i.e., the DNA structure. Indeed, a good precision in the volume of its constituents and their relative position has a direct impact on the number and complexity of damage that is computed from both the physical and chemical stage. This structure has to be defined as precise as possible at the nanometric scale (nucleotide scale) as well as at higher scale (micrometric) where the DNA structure is also meaningful (chromatin fiber, domains, chromatin compaction depending on the cell cycle, etc). Historically, the DNA geometrical models that were implemented range from simplified representations that were based on cylinders [115] to highly complex descriptions of the whole genome [9,116,117], reaching, in some cases, an atomistic definition of the DNA components [118,119]. In general, inelastic collisions are assumed to cause strand breaks depending on the amount of energy that is deposited in the sensitive sites, including sugar phosphate backbone and DNA hydration shell. This amount of energy is, to a certain extent, a modifiable parameter that is specific to each code. The physico-chemical and chemical stages are also crucial since it should be noted that around 70% of the damage from low LET particles is due to indirect effects. It is, therefore, generally considered that only a certain fraction of the simulated hydroxyl radical interactions with the deoxyribose moiety cause DNA strand breakage. Moreover, it is generally accepted to limit the duration of the simulation of the chemical step which makes it possible to account for the scavenging effect of the cellular environment. From this perspective, the results of several MC track-structure codes that were developed for modelling cellular radiation response have been historically (and successfully) compared to experimental data mainly in terms of DSB yields or chromatin fragments [35,39,114].

### 5.2. “FullSim” Complete Simulation Chain for DSBs Calculations

In 2017, Geant4-DNA was used for the first time for simulating the combination of physical, physico-chemical, and chemical stages for the assessment of early radiation damage in terms of DSB yield and complexity at the scale of an entire human genome (fibroblast cell) [116]. One of the key elements of the simulation is the sampling of energy deposition and radical reactions leading to damage in a DNA geometry which must, therefore, be as realistic as possible. The DNA models that are used within this simulation chain are built with the DnaFabric software [120]. This independent software was designed to facilitate the generation of DNA geometries at different scales ranging from the nucleotide pair to the entire genome of any cell type [121] and respecting the different compaction levels. Once the geometrical model is generated, a text file is written that can be read by the DetectorConstruction class of the Geant4-DNA simulation chain. 

**Figure 4:**
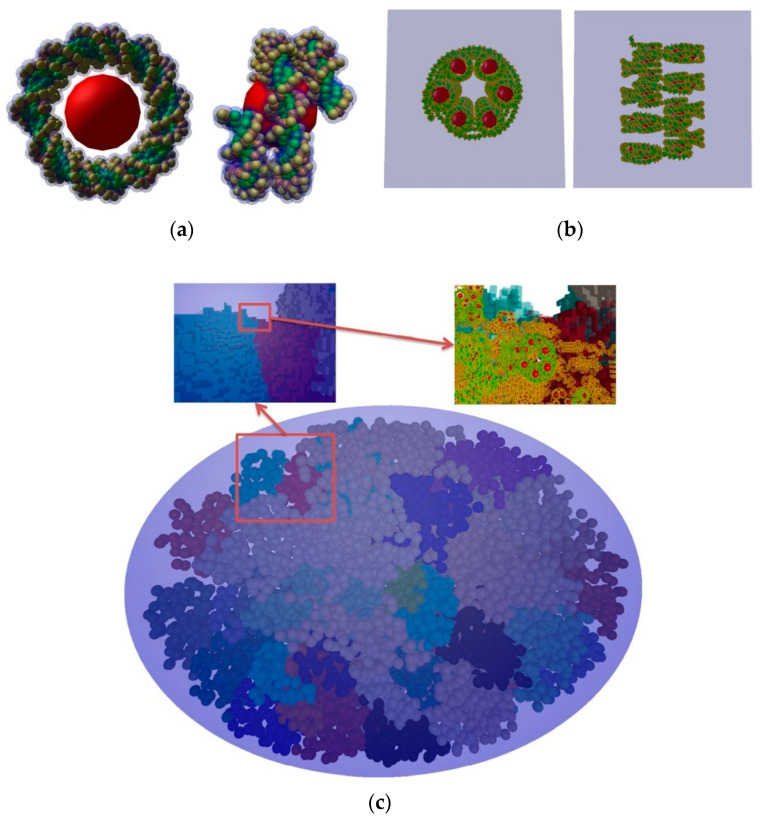
Construction with DNAFabric software of: (**a**) a nucleosome made of B-DNA twisted around a histone, (**b**) a voxel representing a straight voxel of heterochromatin, and (**c**) a fibroblast cell nucleus with different levels of details, taken from [116].

With DnaFabric, B-DNA, which is the most common form of DNA, is modelled by representing each nucleotide constituent (2-deoxyribose, phosphate, and DNA bases) of the genome with spherical shapes and twisted to generate nucleosomes that are linked together to create the chromatin fiber as shown in Figure 4. The continuity of the fiber within each chromosome is ensured by using voxels describing the 30 nm chromatin fiber taking different directions that can be connected to fill the space. From the biological point of view, different DNA densities and distributions are observed at the cellular (microscopic) level depending on the cell type and the cell cycle. Furthermore, chromatin compaction (nanometric level) has a role in the protection of DNA from damage induction and it was shown that DSB production occurs more frequently in decondensed chromatin [122,123]. The condensed form of the chromatin fiber is the heterochromatin whereas the decondensed form is called euchromatin.

Heterochromatin fiber voxel models were first developed in DNAFabric [116] and then complemented by euchromatin models [124]. The combination of the two types of compaction allowed for the accounting of the distribution of heterochromatin and euchromatin regions that were measured experimentally to study its influence on the DSB yield that was simulated for proton irradiations. Results have shown the DSB yield increasing when geometrical models that are closer to the observation are taken into account [124] thus verifying the protective role of heterochromatin which is reproducible in the calculation chain.

Another key element in the simulation chain is the definition of parameters, as shown Table 4, that allow the translation of the simulated information (position of energy deposits and reaction of radicals in the DNA geometry) into strand breaks that are then converted into DSB data. The set of parameters that are used by default to compute the number of DSBs includes: (i) a threshold for energy that is deposited in the backbone (including hydration shell) E_lower_ = E_higher_ = 17.5 eV to induce a direct strand break, (ii) an effective rate P_OH_ = 40% in SSB production from the reaction of a hydroxyl radical with deoxyribose to produce an indirect strand break, (iii) a duration of the chemical step fixed to T_chem_ = 2.5 ns (use of chemistry module of Gean4.10.1) to prevent radicals far from the DNA structure from being at the origin of indirect effects, and (iv) a DSB is scored when at least two SSBs that are located on opposite strands are separated by less than 10 bp. 

The comparisons between the simulated results and the experimental data have been carried out for both high and low LET particles.

**Figure 5:**
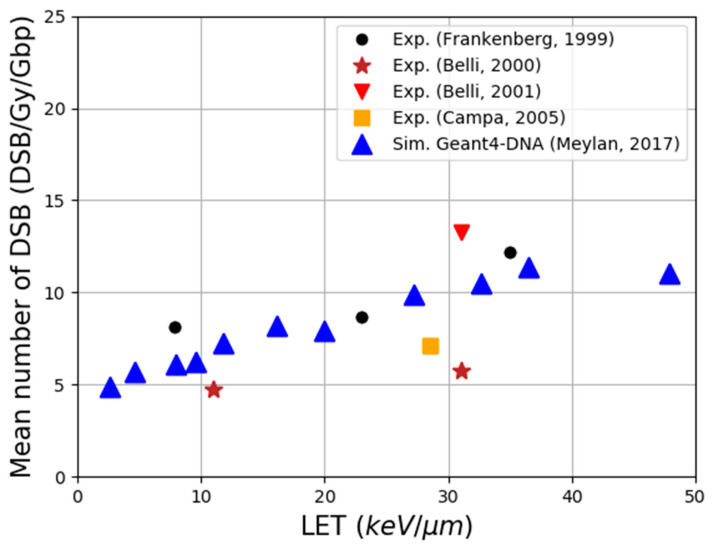
Comparison of DSB yields from the literature with those that were calculated with Geant4-DNA for protons of different LET, reproduced from [116].

DSB yields were first calculated in a fibroblast cell nucleus that was filled with only heterochromatin and compared to the literature for proton irradiations [116]. The good agreement that was found (Figure 5) after fixing the parameters that are presented above, showed that the whole simulation chain was able to reproduce the trend over LET of experimental data, taking into account the measurement uncertainties correctly. A good agreement was also found in a second publication comparing the simulated DSB yield that was produced by X-rays of different spectra (40 kVp, 220 kVp and 4 MV) with the experimental measurements of γ-H2AX for HUVECs cells that were irradiated in the simulated facilities as shown Table 5 [125]. The simulation setup included the nucleus geometry combining heterochromatin and heterochromatin regions that were distributed randomly but respecting the global rates that were observed experimentally. Furthermore, simulations have shown that the differences between different beam qualities were due to the proportion of secondary electrons of energy below 10 keV, highlighting the crucial role of these particles. Since the simulation chain does not take repair processes into account, it is essential to compare simulations and experiments at the maximum gamma-H2AX signal, as suggested in [125], to minimize the underestimation of early DNA damage. Nevertheless, considering the rather slow repair of DSBs, a better match is expected in the case of low LET irradiations because the correspondence 1:1 DSB/foci is more reliable. Indeed, for high LET irradiations, the measured foci may be sites containing clustered DSBs.

### 5.3. Review of “MolecularDNA”Application

An application named “molecularDNA” was developed for the mechanistic modeling of radiation track-structure effects and to test their ability to model the induction of cellular damage following irradiation, including, for the first time, the IRT approach. This application linked the physical, chemical (IRT), and geometrical interfaces of Geant4 to investigate early DNA damage that was induced by radiation and the subsequent biological response. The development and main principles of the application are described in [126], followed by a series of publications [71,106,117,127,128] and is designed for general purpose investigation of how DNA is damaged in a variety of biological geometry models. It contains a library of geometrical models of sub-cellular component units such as simple DNA fibres, chromatin fibres, and fractal geometries that can combine smaller segments to build a longer chain. The molecularDNA application uses the damage classification system that was proposed by Nikjoo [129], which allows the calculation of the number of initial DNA damage events for a given simulation. The classification system considers the source of the damage (direct and indirect damage induction) and the complexity of the damage, including simple SSBs, DSBs, and complex clustered damage. By using the scored initial DNA damage and the classified damage detail as inputs, the application allows calculation of the protein yield that is involved from the four major repair processes in normal human fiblobrasts. 

**Figure 6:**
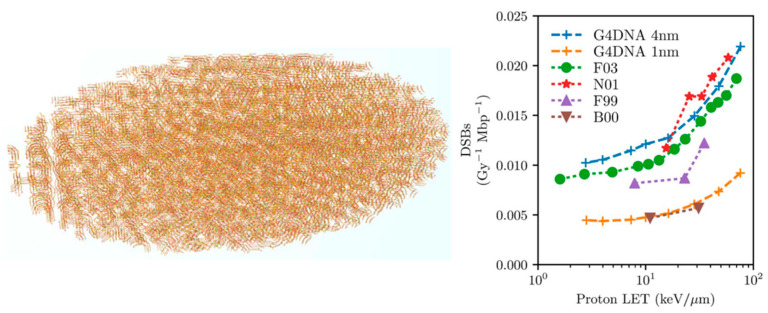
Left: *E. coli* geometry that was generated by the molecularDNA application. Right: the DSB yields as a function of LET from proton irradiation assessed by Geant4-DNA, compared to the results of PARTRAC (F03) and KURBUC (N01), and experimental data (F99, B00). Taken from Lampe et al. [71].

The application was validated through a comparative investigation of Geant4-DNA physics, chemistry, and DNA damage models against some well-established MC simulation platforms for radiobiological applications [117], namely KURBUC and PARTRAC. For the assessment, a 3 μm radius liquid water sphere was filled with 200,000 randomly-distributed, non-overlapping, individual DNA geometries. Each DNA geometry was a 216 base-pair (bp) straight DNA segment in a rectangular placement volume. Primary electrons (energy range of 300 eV to 4.5 keV) were generated randomly with a random direction in a smaller 500 nm radius sphere in the centre of the test region. In a follow-up study, the application was extended to allow the simulation of initial DNA damage in an *Escherichia coli* (*E. coli*) cell using a combination of straight and turned DNA segments [71] that were joined together to mimic a fractal pattern. Applying a mask to this otherwise rectangular pattern allowed simulation of the *E. coli* ellipsoidal geometry as shown in the left panel of Figure 6 (a long semi-major axis of 950 μm and two short semi-major axes of 400 μm) containing 4.63 Mbp. The simulated results are validated against experimental data for plasmid that was irradiated by both electrons (10 keV) and protons (90–249 MeV), as well as against past simulations. Through the above two studies, the damage models and parameters were tuned to be consistent with experimental data and the simulated result of KURBUC. For direct damage, both a threshold model (where breaks occur when energy depositions are E_lower_ = E_higher_ = 17.5 eV or higher, proposed by KURBUC [129]) and proportional damage model (where breaks occur with a linearly increasing likelihood from E_lower_ = 5 to E_higher_ = 37.5 eV, proposed by PARTRAC [36]) were tested for different energy deposition ranges. The probability of indirect damage (*P*_OH_) was also tested within the ranges for which it had been experimentally measured (0.4–0.8) [130,131]. As a result of this tuning, the Geant4-DNA simulations that were reproduced the results of KURBUC with similar parameters used in KURBUC as shown in right panel of Figure 6.

**Figure 7:**
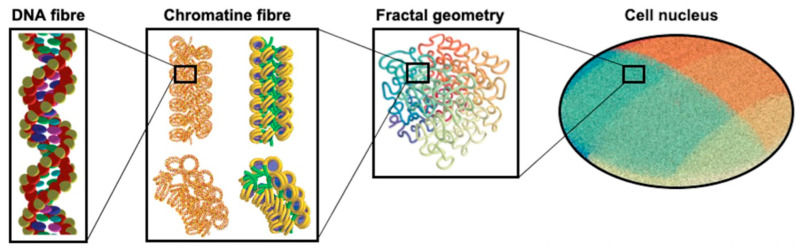
The structure of cell nucleus and its sub-biological components for the molecularDNA application [128].

The geometrical model was further improved in the study of Sakata et al. (2019) [127] to build a human cell nucleus that was composed of fractally distributed chromatin fibres as shown in Figure 7. In this newly developed cell nucleus model, the DNA fibre is folded by histones (which were defined as spheres, each with a 25 Å radius) compactly forming chromatin fibre. In the study by Sakata et al. [127], the damage parameters were re-adjusted within a reasonable range to achieve agreement with experimental data for proton irradiation that was induced SSB and DSB yields in a human cell.

**Figure 8:**
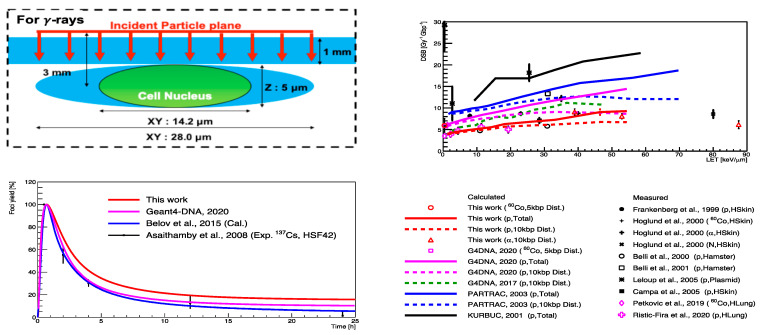
(**Left-top**): The geometrical configuration for a human cell from Sakata et al. [128]. Right: the DSB yields as a function of LETtaken from Shin [106]. (**Left-bottom**): the yield of γ-H2AX as a function of time after irradiation taken from [106].

In reality, a cell nucleus is confined to a cytoplasm and irradiation events involve the surrounding experimental equipment. Thus, in the follow-up study by Sakata et al. [128], the geometrical model was upgraded with an ellipsoidal water absorber to mimic the cytoplasm and a rectangular absorber to imitate the window of a cell culture flask (shown in the left-top panel of Figure 8). Additionally, the model parameters were slightly adjusted, and a prediction model that was given by Belov et al. [132] that calculates kinetics of proteins that are involved in four major repair pathways for normal human fiblobrasts was integrated into the application. The fully integrated application was validated with both the experimental DSB yields (as Geant4-DNA 2020 in Figure 8, from [128]) and accumulated γ-H2AX yields. Finally, the influence of the updated geometrical model and the optimal parameters that were proposed with the recent improvements in physical and chemical stages [11,133] were evaluated (see Table 4) and validated in [106] for both gamma- and proton-irradiations (‘this work’ in the left and right panels of Figure 8). 

The “molecularDNA” application provides a fully integrated simulation chain consisting of the physical, chemical (IRT), and biological stages of irradiation at the sub-cellular scale. This application has been fully validated through a series of Geant4-DNA investigations and the simulation results of DSB yields for a human fibroblast cell are in good agreement with experimental data. The “molecularDNA” application will soon be released in Geant4 and can be used for understanding the mechanisms leading to cellular radiobiological effects.

## 6. Geant4-DNA Extended Examples

As discussed in the previous sections, Geant4-DNA provides functionalities for the simulation of the interactions of ionizing radiation in liquid water as well as the modeling of pre-chemical and chemical stages of water radiolysis that can be combined with simplified models of biological cellular and sub-cellular targets for damage and repair prediction. However, like Geant4, Geant4-DNA provides a set of computing libraries, and their use requires a minimum knowledge of C++ coding. Therefore, to help users understand the functionalities and develop new applications, the Geant4-DNA collaboration has developed a set of examples that are located in the “extended/medical/dna” category of the Geant4 examples that present the usage of processes and models covering from physical interactions to the chemical stage including simplified biological geometric models. A recent publication that was dedicated to track-structure simulations in liquid water medium describes these examples and their main objectives [23]. Interested readers are encouraged to consult the associated references for a detailed description of each particular application. Beyond their pedagogical role, these examples also allow the verification and validation of Geant4-DNA simulations against literature data or international recommendations as well as regular Geant4 regression tests being performed to test each new Geant4 release [23,134,135]. These examples are maintained and updated along with the Geant4 bi-annual releases. They are briefly described in the following subsections and categorized according to the stage of radiation action they serve.

### 6.1. Physics Examples

These Geant4-DNA examples are directly related to the main transport and energy deposition magnitudes. In each of these examples, the irradiated geometry and physics list can be modified. In many cases, condensed-history physics models can also be enabled.

- The “*clustering*” example calculates the energy deposition with a dedicated clustering algorithm to assess DNA strand breaks in a simple liquid water geometry [14];
- “*dnaphysics*” is a general example that enables track-structure simulation of charged particles in a liquid water geometry and allows for the automatic combination between Geant4-DNA physics models and condensed-history models at higher energies (i.e., above 1 MeV) and can be used for benchmarking simulations that are related to track-structure characteristics [23];
- “*icsd*”, that stands for ionization cluster size distribution, calculates the number of ionizations for each simulated track in a cylinder mimicking a piece of chromatin and uses DNA-like material’s cross sections that were obtained experimentally or by simulations [50];
- “*mfp*” stands for mean free path and allows the calculation of the aforementioned distance and related distance quantities for a charged particle in a sphere geometry of liquid water [23];
- “*microdosimetry*” simulates lineal and specific energy distributions and related quantities in liquid water spheres that are randomly placed along the particle track [59];
- “*microprox*” is another microdosimetric example that calculates proximity functions from energy depositions scored in liquid water spherical shells from randomly selected hits [60];
- “*range*” example performs a simulation of penetration distances in liquid water [70];
- “*slowing*” enables simulation of the slowing down spectra of electrons in a cube of liquid water [136];
- “*splitting*” uses variance reduction techniques to improve the efficiency of the calculation of ionization cluster size distributions. This is done in a nm sized cylinder as in the case of the icsd example and aims to separate secondaries that are generated within the cylinder to avoid the overlapping of tracks [137];
- “*spower*” allows for stopping power simulations of particles in liquid water with the use of specific physics modules that enable the use of a stationary mode for appropriate computation [23];
- “*svalue*” calculates the dose to a target volume per unit of cumulated activity in a source volume, called S-value [138,139]. The source and target volumes can be different cell compartments or an entire cell of a simple spherical geometry which can be modified to account for more complex cell geometries, as has been done in many studies i.e. [140,141];
- “*wvalue*” serves to simulate the mean energy that is expended to form an ion pair known as W-value. It also provides information on the total number of ionizations in a liquid water volume and its penetration details. It is a useful benchmark simulation for the inelastic models given that elastic interactions are indifferent in this simulation scheme [23,58].

### 6.2. Chemical Examples

The”chemX” examples provide the guide for the chemical module from the activation of the chemical stage to the calculation of radiochemical yields (“G-value”) as a funtion of time. In each example, irradiated geometry is a large water volume and as in the case of physics examples, the physics list can be modified.

- “chem1” aims to show how to activate or deactivate physicochemical and chemical stage after physical stage. Chemical reactions are printed and the step-by-step model is used by default.
- “chem2” provides a user-class “TimeStepAction” which allows users to change Minimum Time Steps. These parameters constrain the minimum time-step that is allowed for each reactant pair using the step-by-step model. The user-class also shows how to print reaction information such as reactants and products as well as their positions.
- “chem3” illustrates how to implement user actions in the chemistry module using the step-by-step model. Users can also visualize the trajectories of the chemical species in time and space using the graphical user interface.
- “chem4” provides scorer classes to compute radiochemical yields (“G”) versus time using the step-by-step model, including a dedicated ROOT graphical interface. The G-value is useful for benchmark simulations in comparing with other MC codes and experimental data [80].
- “chem5” computes radiochemical yields (“G”) versus time using alternative physics and chemical reaction lists using the step-by-step model [142].
- “chem6” computes radiochemical yields (“G”) versus time and LET using the IRT model with full macro control [11,104].

### 6.3. The dnadamage1 Example

Following the “FullSim” simulation chain that is presented above [116], the recent Geant4-DNA example *dnadamage1* method has been released since Geant4.10.6. In this example, we placed a cubic volume of 40 × 40 × 40 nm^3^ at the center of a 2 × 2 × 2 µm^3^ box made of liquid water (1 g/cm^3^). Inside the 40 × 40 × 40 nm^3^ cubic volume, 3640 nucleotide pairs were built to form a piece of 40 nm heterochromatin straight fiber. This geometrical DNA model was generated with the DNAFabric software. More information about the generation of this geometrical model can be found elsewhere [120]. The total number of strand breaks is computed from the combination of direct and indirect damages. Concerning the physical stage leading to direct damage, the default G4EmDNAPhysics is used. Direct damages are scored if the cumulative deposited energy from ionizations and excitations in the individual volumes of a nucleotide backbone (i.e., the volumes that are representing a group of the phosphate, the 2-deoxyribose, and the hydration shell) is greater than 17.5 eV. For the chemical stage, the G4EmDNAChemistry_option2 constructor is then used to simulate the species diffusion and their reactions with each other or with DNA elements (phosphate, the 2-deoxyribose, and base pairs) using the current SBS model [79]. A reaction between OH•radicals and static DNA elements is counted as primary damage. When one reaction happens, the radical is killed and the damaged DNA element is no longer available for further reaction. It has to be noted that simulated damage is primary damage that is transformed into a SSB with a probability of 42% [116]. More details about the C++ classes and their structure can be found through the README file of the example. To improve the computation time of the example, the IRT method is currently implemented in this example as an option [105].

## 7. Conclusions

MC track-structure codes are capable of providing both the spatial pattern of the energy deposition within a medium as well as the details of the molecular modifications that are taking place after irradiation. As of this moment, this has not been achieved experimentally. The value of such MC codes in elucidating the mechanisms of cellular damage from ionizing radiation is beyond doubt. The spatial distribution of the interaction events dictates the proximity of DNA strand breaks and, at the same time, the alterations that are taking place in the target molecule(s) determine the chemical modifications. It must always be stressed that all the results that are obtained with the MC technique are as accurate as the input information (e.g., the interaction cross sections for the physical stage of radiation action). 

The Geant4-DNA low energy extension that was developed for biomedical applications is constantly evolving in terms of the development of physics models towards the accomplishment of a realistic cellular environment subject to irradiation conditions. Currently, it offers different sets of physics models, chemistry modules, and cell geometries. The combination of the above has allowed the realization of mechanistic studies of cellular DNA damage and repair. Extension of the Geant4-DNA Option 4 track-structure model for electrons up to 10 MeV is underway as well as two alternative models for protons up to 300 MeV. Furthermore, material that is specific cross sections for biopolymers [51] and for gold [143,144] that are used in nanodosimetry have already been developed and tested and they are soon going to be made available to the scientific community through Geant4-DNA. Especially, the cross sections for gold can contribute significantly to the gold nanoparticle-aided radiotherapy research [145,146]. The modeling of the chemical stage is currently being improved [133] and extended to longer times and macroscopic volumes [147]. This is a requirement for easier comparison with radiolysis experiments, which also paves the way to the simulation of radiolysis in FLASH radiotherapy conditions, currently a very active research topic. A library of multi-scale geometries from molecules to assemblies of cells that are compatible with Geant4-DNA physical and chemical interfaces, will also be made available to users in the near future, and efforts to improve the computational performance of Geant4-DNA will continue. All these developments will permit a wider range of radiotherapeutic applications of the code under different irradiation scenarios, to be studied in an in silico, bottom-up approach.

The activity on DNA damage and response is intense within the Geant4-DNA framework and important studies have been published that involve mechanistic studies of DNA damage taking into account details of chemistry [11,71,104,128,133]. Such work will continue towards the goal to connect the irradiation of a cell environment to the DNA response to damage and repair. The ultimate goal is to offer the scientific community all the state-of-the-art tools to study the radiation effects in DNA and cells and to illuminate differences in RBE for low and high LET beams which is a matter of importance, not only for radiotherapy purposes, but also for space radiation risk studies and radiation protection low dose issues. It is envisioned that Geant4-DNA will offer a complete open-source platform available in Geant4 that is able to simulate physical, physicochemical, chemical, and biological processes that are occurring after irradiation of human cells. Further work along these lines is ongoing and will be published in future publications.

## Figures and Tables

**Table 2:** Default decay channels and corresponding branching ratios of a water molecule until 1 ps as currently available in Geant4-DNA (the symbol * is used to represent excited water molecule).

Electronic State	Decay Channel	Fraction
All ionization states	$H_{2}O^{+}+H_{2}O\;\to$H_3_O^+^ + ^•^OH (through proton transfer)	100%
Excitation state A1B1: (1b1) →(4a1/3s)	$H_{2}O*\to \;$^•^OH + H^•^ $H_{2}O*\to \;$H_2_O + ∆E	65% 35%
Excitation state B1A1: (3a1) →(4a1/3s)	$H_{2}O*\to \;$HO^+^+ ^•^OH + e^−^_aq_ $H_{2}O*\to \;$^•^OH + ^•^OH + H_2_ $H_{2}O*\to \;$H_2_O + ∆E	55% 15% 30%
Excitation state: Rydberg, diffusion bands	$H_{2}O*\to \;$HO^+^+ ^•^OH + e^−^_aq_ $H_{2}O*\to \;$H_2_O + ∆E	50% 50%
Electron attachment	$H_{2}O^{-}\to \;$OH^−^ + ^•^OH + H_2_	100
Electron-hole recombination	$H_{2}O*\to \;$^•^OH + H^•^	55%
	$H_{2}O*\to \;$H_2_ + 2^•^OH	15%
	$H_{2}O*\to \;$H_2_O + ∆E	30%

**Table 3:** Implemented chemical reactions and reaction rate constants k [82,83] as defined in the two chemistry constructors “G4EmDNAChemistry” and “G4EmDNAChemistry_option1” that apply at ambient temperature (25 °C) of Geant4-DNA version 10.7.

Reaction	$\mathrm{Reaction}\;\mathrm{Rate}\;\mathrm{Constant}\;k\;(10^{10}M^{-1}s^{-1})$	
	G4EmDNAChemistry	G4EmDNAChemistry_ Option1
$e_{aq}^{-}+e_{aq}^{-}+2H_{2}O\to H_{2}+2OH^{-}$	0.5	0.636
$e_{aq}^{-}+H{}^{•}+H_{2}O\to H_{2}+OH^{-}$	2.65	2.5
$e_{aq}^{-}+{}^{•}OH\to OH^{-}$	2.95	2.95
$e_{aq}^{-}+H_{3}O^{+}\to H{}^{•}\;+H_{2}O$	2.11	2.11
$e_{aq}^{-}+H_{2}O_{2}\to OH^{-}+{}^{•}OH$	1.41	1.10
${}^{•}OH+{}^{•}OH\to H_{2}O_{2}$	0.44	0.550
${}^{•}OH+H{}^{•}\to H_{2}O$	1.44	1.55
$H{}^{•}+H{}^{•}\to H_{2}$	1.2	0.503
$H_{3}O^{+}+OH^{-}\to 2H_{2}O$	14.3	11.3

**Table 4:** Main parameters of FullSim and MolecularDNA simulation chains for predicting early DNA damage.

Parameters		FullSim	MolecularDNA
Physical parameters	R_dir_ (Å)	VDWR + hydration shells *	3.5
	E_lower_(eV)	17.5	5.0
	E_higher_(eV)	17.5	37.5
Chemical parameters	P_OH_	0.4	0.405
	T_chem_ (ns)	2.5	5.0
	d_kill_ (nm)	N/A	9.0

R_dir_: Accumulation radius of energy deposition from nucleotide centre. E_lower_: Minimum energy of direct strand break probability model. E_higher_: Maximum energy of direct strand break probability model. P_OH_: Probability of indirect strand break. T_chem_: Time limit of chemical diffusion. d_kill_: Production range limit of chemical radiolysis species from nucleotide centre. (VDWR): Summing up of atomic volume with each atomic van der Waals Radius (1.2, 1.7, 1.5, 1.4, 1.9 Å for H, C, N, O, P respectively). (*) Additionally, 24 water molecules considered as hydration shell.

**Table 5:** Comparison of the simulated results and the experimental for a dose of 1 Gy for different X-ray beams: mean number of γ-H2AX foci per endothelial cell nucleus in Gap0/Gap1 (30 min post-irradiation) and mean number of simulated DSB per nucleus, reproduced from [125].

Simulated DSBs and Experimental Foci at 1 Gy	40 kVp X-rays	220 kVp X-rays	4 MV X-rays
Sim. mean number of DSBs per nucleus	21.0 ± 0.3	21.0 ± 0.3	16.8 ± 0.3
Exp. mean number of γ-H2AX foci per nucleus	18.59 ± 0.43	18.64 ± 2.33	16.46 ± 1.63
